# A study of the roles of school administrators in increasing the quality of school life through social responsibility projects in primary schools

**DOI:** 10.3389/fpsyg.2022.969638

**Published:** 2022-09-27

**Authors:** Aşkın Doygunel, Fatma Koprulu

**Affiliations:** Department of Educational Administration and Supervision, Near East University, Nicosia, Cyprus

**Keywords:** primary education, school administrator, social responsibility, social responsibility projects, quality of school life

## Abstract

The structure and expectations of societies are constantly changing, developing, and advancing as time demands. Accordingly, the vision, mission, purpose, and objectives of educational institutions are changing and are shaped according to the expectations of the society. School Directors, teachers, and families, briefly the community, should know that schools are institutions that best fulfill children’s learning, and make them feel happy and safe. A cheerful and peaceful school environment always brings academic success. Children who have a quality school life are aware of the responsibility for their behaviors as well as the social responsibilities for their environment. Thus, students who are closely interested in social responsibility projects are not just academically developed; at the same time, their social, emotional, and mental development increases, and their social cohesion and awareness develop. This research aims to examine ways to improve the quality of social life in schools through social responsibility projects to be started as well as opinions by School Directors. In this study, the qualitative method and case study design were used. The study group of this research consisted of 15 teachers working in primary schools affiliated with the Department of Primary Education of the Ministry of National Education. The participants were interviewed through live connections. The findings revealed that students are very willing to take part in social responsibility projects, and significant improvements have been made in their academic achievement; they attended more willingly, and there have been noticeable changes in the quality of the school.

## Introduction

Education is a multifaceted process consisting of wide, complex, and diverse teaching and learning levels. Therefore, in addition to the courses processed in the classroom, the behavioral changes that are desired to be created in the student can be achieved in different and various ways. Since education is a whole, it is very important to develop effective and emotional behaviors such as helping, sharing, cooperating, and the students’ academic development. For this reason, the school administrators should be well aware of their educational leadership and offer forward activities. In addition to academic achievements, the development of students’ developmental behavior is reflected in the quality of school life. Special emphasis should be placed on the quality of school life by administrators and teachers. Yılmaz (2005) emphasized it is crucial to examine the quality of school life, students’ interests and expectations, their reactions toward their teachers, and their commitments. The behaviors and characteristics that society expects to see in individuals, especially in primary school students, will only be adopted through good management and healthy organization. To have all the academic, social, physical, mental, and developmental characteristics necessary for the development and success of students, the main task in planning and conducting research in this field falls to the school administrator. Thus, in addition to academic knowledge, well-planned and managed social activity studies are also important in contributing to students in all areas (Kir, 2013).

Social activities, which have an important place among the tasks of educational institutions, are shaped by the desire and perseverance to work within the teacher, which arouses students’ interest and desire. The responsibility taken by the student and the happiness in fulfilling this responsibility shows how important social activities are in his/her life (Yılmaz, 2007).

Giving students a say in the functioning of the school also leads to more efficient social activities. The quality and importance of the communication that the school administrator provides with students and the opportunities that the school provides for the student affect the students’ commitment to the school (Yüksel, 2012).

Suh and Traiger (1999) stated that the importance and necessity of both parental and social responsibility arose when children were given personal responsibility education in schools and that the school curriculum should support moral decisions and parents’ value education (Gündüz, 2018).

Extracurricular activity studies have an important place in the student’s life. With such extra-course activities, there are good developments in the behavior of students, there are forward increases in school performance, there are noticeable decreases in school absences, socialization is ensured in all directions, and they are effective in becoming adult’s worthy of their family and society. Therefore, school administrators should be planners, routers, and guides and make teachers realize, encourage, and support how important such activities are in education (Filiz and Nayir, 2015).

It is important for students to experience and develop citizenship qualifications, which include various skills and qualities such as voluntary behavior, empowerment, civic activity, and critical thinking, which indicate community benefit, cooperation, and non-reward, both in the classroom settings and in communities where this environment is expanded (Coşkun, 2020).

With this research, an answer was sought to the question “What are the opinions of school administrators about increasing the quality of school life through social responsibility projects in primary schools?”

With this research, an answer was sought to the question “What are the opinions of school administrators about increasing the quality of school life through social responsibility projects in primary schools?”

The results of this research are as follows: In primary educational institutions, it is thought that “what extra-curricular activities are, how the students’ desire to participate in these activities is, what are the positive and negative behaviors that extra-curricular activities bring to students, will contribute to the area where the future of the students will be bright and will be satisfactory for families and school.” To achieve this goal, answers to the following interview questions were sought.

1. As a primary school administrator, what do you think about the “quality of school life?”

2. What duties are assigned to the school administrators in terms of increasing the quality of school life?

3. What projects can be carried out and what are their roles in social responsibility issues?

4. What do you think should be done to increase social responsibility projects in primary schools?

5. Does the increase of social responsibility projects in primary schools have an impact on the quality of school life?

## Literature review

As mentioned above, there are a large number of researches relating to social responsibility projects. Nevertheless, few of these researches have been concentrated on social responsibility project in schools. For schools which is our interest, Tuzcu and Savaşkan (2020) stated that social responsibility projects not only supply social outcome but at the same time provide individual and organizational achievements. Cetindamar and Hopkins (2008) underline that the integration of social responsibility into education is a daunting—and rewarding—task of assisting students in understanding diverse values and gaining action skills. Pozo et al. (2016) in their research titled “*Teaching personal and social responsibility model-based programmers in physical education. A systematic review*” pointed out that when students are offered the right strategies and skills, they will be more responsible in their daily lives, both in and out of the school context. Selvi and Şentürk (2016) aimed to explain the “*Social responsibility approach in civil society organizations.*” The study stressed that social responsibility is not just related to a single industry. It is a notion that affects all classes of society. Social responsibility projects are planned to raise awareness of environmental and social issues in our children, who are the future of our country, to find solutions to problems, and to help children develop themselves and carry out social responsibility projects (Uğurlu and Arslan, 2015).

Toma (2006) reported that “quality” has a close relationship with social responsibility and is a widely applied concept, targeting both products and services and the environment, and life in general. Busher et al. (2015) stated that the essential task for schools is to guide students to achieve the planned goals. Thus, an effective and successful school can be defined as students having achieved the goals planned for them. Seçer and Sari (2006) claimed that social responsibility activities play an essential role in students’ life. Thus, if the school guides the students to join these activities, the students will be happier and more willing to go to school.

In light of the above research, it indicates once again that social responsibility projects are essential in human life. Moreover, they increase the quality of school life.

## Methodology

In this section, the topics of research pattern, study group, data collection process, data collection tool, and data analysis are discussed. The qualitative research method was administered to determine the views of school administrators on “the roles of school administrators in increasing the quality of school life through social responsibility projects in primary schools.” One of the qualitative research patterns, “the case study,” was used to collect data for the study (Yin, 2011). The case study approach is particularly useful to employ when there is a need to obtain an in-depth appreciation of an issue, event, or phenomenon of interest, in its natural real-life context (Crowe et al., 2011). In addition, the semi-structured interview technique from qualitative research methods was used in the study.

### Data collection procedures

After obtaining the necessary permits from the Ministry of Education of the Turkish Republic of Northern Cyprus, face-to-face interviews and connections were conducted with the participants between 25 October and 5 November 2020. They were briefed on all the rules well in advance and were assured about the purpose of the study and confidentiality. Each of the interviews lasted about 35 min. The participants were asked five open-ended questions, and their responses were noted down on interview forms.

The questions presented to the participants in the interview form were written in clear, simple, understandable, and proper Turkish. The Interview Form is called “Views about the Roles of School Administrators in Improving the Quality of School Life through Social Responsibility Projects in Primary Schools.” The form consists of two parts. The first part includes demographic (personal) information about school administrators, while the second part contains interview questions that will reveal the purpose of the research. Pilot applications were also carried out with several school administrators to control these questions.

### Data analysis

The data obtained were first conceptualized and organized logically. Then, themes were created to analyze the data using the content analysis method (Downe-Wamboldt, 1992). Based on exploring themes and codes, the researchers have created their groups and themes and they also resolved the data and encoded it. The participants were codified as A1, A15 etc. (A referring to Administrator). The encodings were separated and placed under the themes. Finally, frequency and percentage tables for encodings were created.

The participants were 15 primary school administrators in the Turkish Republic of Northern Cyprus, Ministry of National Education in the 2020–2021 academic year.

The researchers’ role in this study serves as an intermediary between the participants and the data that was being collected. Also, the researchers’ role is to analyze the information in detail and construe the hypothesis. The focus group that was interviewed in this study are primary schools’ administrators in North Cyprus. In this research paper, the data were collected from 25 participants who are well experienced and responsible in their present job.

### Limitations of the study

This research is limited to public and private primary schools in six different districts of the TRNC between the 2019 and 2020 academic year, and the working group is limited to 25 primary school administrators. Finally, for document analysis, the pictures are obtained before COVID-19.

As can be observed in Table 1, the 15 primary school administrators are considered to be young, between the ages of 35 and 45.

**TABLE 1:** Demographic characteristics of the participants.

Number	Gender	Age	Length of service	Length of service in administration
1	E	37	15	3
2	K	45	23	8
3	E	41	19	6
4	K	40	18	6
5	E	38	16	4
6	K	43	21	7
7	K	44	22	9
8	E	36	14	4
9	E	39	17	8
10	E	40	18	4
11	K	37	15	3
12	E	38	16	5
13	K	42	20	9
14	K	36	14	3
15	E	35	13	2

### Findings

The findings of the study were revealed based on the answers to the five interview questions asked to the participants. Six themes were found in the study, and the findings of these themes were tabled in frequency and percentage as presented below.

When Table 2 is examined, it can be seen that the views by the school administrators about the “quality of school life” are classified under 109 codes and 11 themes. Fifteen of the views expressed school life as “a place where teachers, students, and all the staff are happy,” 14 as “good level of education and training,” 13 as “teachers are successful and qualified,” 11 as “a safe structure and school environment in all directions and an institution where socialization takes place,” 10 as “social activities are at the forefront,” 9 as “have a disciplined school environment,” 7 as “a technologically advanced structure,” 6 as “a clean and orderly environment,” and 5 as “good relations with the Ministry of Education.”

**TABLE 2:** Administrators’ views about the “quality of school life.”

Theme	Frequency (f)	Percentage (%)
A place where teachers, students and all the staff are happy	15	13.7
Good level of education and training	14	12.8
Successful and qualified teachers	13	12
Safe structure and a school environment in all directions	11	10.1
Institution where socialization takes place	11	10.1
Social activities at the forefront	10	9.2
A disciplined school environment	9	8.3
An environment where all developmental characteristics of students are improved	8	7.3
Technologically advanced structures	7	6.4
Clean and tidy environment	6	5.5
Good relations with the Ministry of Education	5	4.6
**Total**	109	100

I believe that school administrators should not be in the latest place in their relationship with the Ministry of Education when linking the “quality of school life” more to the fact that the school is seen as a happy nest that education and training are good and that school teachers are successful and qualified. Because the Ministries of Education have a very important place in the quality of the schools. Some of the administrators’ views are as follows:

*“According to me, a qualified school represents a home where teachers, students and all the staff are happy. It also shows a structure in which students socialize” (A1)*

*“In my opinion, it creates an environment where all developmental characteristics of students are improved. It also expresses a clean and orderly environment, a disciplined structure.” (A6)*

*“It is explained that education and training are at a good level, teachers are successful, qualified, and well aware of the studies carried out.” (A10)*

*“A qualified school reminds me of a quality school structure. The headmaster dedicated himself to education and training all his staff to succeed in all aspects. In addition, it emphasizes social activities for student development” (A10).*

As in Table 3, the administrators’ views about the subject question are classified under 87 codes and eight themes. Fourteen views emphasized good communication with the students, teachers, and the other staff, 13 expressed the task as paying attention to extracurricular activities, 12 stressed the importance of enjoyable educational activities, according to students’ expectations and learning levels and “provide a learning environment, 10 advocated taking students’ opinions into account in some applications,” eight said the diversity of course tools needed to be increased, and seven suggested more physical equipment.

**TABLE 3:** Duties of school administrators in improving the quality of school life.

Theme	Frequency (f)	Percentage (%)
Good communication with students, teachers and all staff	14	16.1
Attention to extra-course activities	13	14.9
Making activities enjoyable according to students’ expectations and learning levels	12	13.8
Providing a learning environment by doing	12	13.8
Attention to students’ social and personal development as well as academic development	11	12.6
Students’ opinions should be taken in some applications	10	11.5
Increasing the diversity of course tools	8	9.2
Providing physical equipment	7	8.1
**Total**	87	100

In my opinion, this ranking should also include the importance of “the close communication and relationships of school administrators with families.” Some of the their views are as follows:

*“For me, in order to create a qualified school, important tasks are assigned by the school administrators in the first place. At all times, the teacher, student, and all the other staff should involve in taking steps and raising ideas.” (A2)*

*“In my opinion, the architect of a qualified school is the school administrator. He should pay attention to extracurricular activities with plans and programs that he will prepare, to make the learning environment enjoyable.” (A7)*

*“In my opinion, the most important task in increasing the quality of school life falls in the school administrator. Educational activities should be made enjoyable to respond to student expectations and learning levels, providing students with a learning environment to do and learn.” (A11)*

*“School Directors should pay attention to the social and personal development of the students as well as their academic development, and in some applications, their opinions should be taken into account to make them feel confident. In addition, provide a course diversity and create an appropriate working environment” (A15)*

As Table 4 reveals, the roles of school administrators are classified under 134 codes and 12 themes. 15 participants advocated awareness of responsibility, 15 emphasized that education is not only academic information, 14 pointed to the importance of planning, schedule, and coordination, 13 said administrators should be models for the staff and students, 12 suggested referring to the views of all stakeholders, 11 stressed encouragement and rewards for teachers and students, 10 stated that social responsibility should be a way of life, nine said schools should get support from family associations and parents as well as from the local government, eight pointed out that schools should get support from the environment, institutions, and organizations, and seven advocated the need for financial and moral support from the Ministry of National Education.

**TABLE 4:** Role of school administrators in social responsibility projects.

Theme	Frequency (f)	Percentage (%)
Instill awareness of responsibility among the staff and especially students	15	11.2
Show that education does not consist solely of academic knowledge	15	11.2
Act planned and programmed, work in coordination	14	10.4
Be a role model for all school staff and especially to students	13	9.7
Consider the opinions and suggestions of all its stakeholders	12	9.0
Encourage	11	8.2
Reward teachers and students	11	8.2
Make social responsibility a way of life	10	7.5
Receive support from the family association and parents	9	6.7
Get support from local governments	9	6.7
Receive support from the environment, institutions and organizations	8	6.0
Receive financial and moral support from the Ministry of Education	7	5.2
**Total**	**134**	**100**

It should not be overlooked that financial support is also needed in the full fulfillment of social responsibility projects. In this study, school administrators considered the need for financial support in the latest plan. There should be sponsors who will provide financial support directly for the project. Some of the administrator views are as follows:

“F*or me, all the staff and particularly the students should be made aware of responsibilities at an early age and should know that education is not only academic knowledge” (A3)*

*“A school director should implement plans and programs in social responsibility projects, work in coordination, and be a role model for all school staff and especially students” (A5).*

*“My opinion is that ideas and recommendations from stakeholders should be considered, encouraged and to increase students’ success in social responsibility projects.” (A8).*

*“We, the school administrators, should firstly assume social responsibility awareness a principle so as to be able to raise awareness among all the involved. We should also point out that education does not only consist of academic knowledge. All the requirements for student development need to be met. Schools should get support from family associations, parents, local governments, the environment and organizations.” (A13).*

As in Table 5, the subject question is classified under 101 codes and nine themes. Fifteen participants are in favor of “charity and solidarity events,” 15 for “health activities,” 14 for “environmental awareness activities,” 12 for “sporting events,” 11 for “educational activities,” 10 for “cultural and artistic activities,” nine for “activities to love and protect animals,” eight for “craft activities,” and seven for “natural disaster prevention activities.” In this respect, he demonstrated with the first three themes that the TRNC is a Nation that is sensitive to its people and cares about the value judgments of its society. Some of the administrator views are as follows:

**TABLE 5:** Social responsibility projects that can be done in primary schools.

Theme	Frequency (f)	Percentage (%)
Cooperation and solidarity activities (to meet the needs of clothing, books and various tools…)	15	14.9
Activities in the field of health (wellness trainings for disabled people, hospitals, nurseries, nursing homes, first aid trainings, hygiene trainings…)	15	14.9
Environmental awareness activities (recycling projects, forest, and reforestation projects…)	14	13.8
Sports activities	12	11.9
Educational activities (developing materials and sending them to needy schools, book reading days…)	11	10.9
Cultural and artistic activities	10	9.9
Activities to love and protect animals	9	8.9
Craft activities	8	7.9
Activities to prevent natural disasters	7	6.9
**Total**	**101**	**100**

*“According to me, cooperation and solidarity activities, activities in the field of health are the social responsibility projects that we face the most frequently” (A1).*

*“If a school adminstrator knows the demographic status of his school and its surroundings well, he will be successful in implementing social responsibility projects. At this point, the importance of environmental awareness and organization of activities should be well understood” (A4).*

*“For me, social responsibility projects should include sports events, educational, cultural and artistic1 activities.” (A9).*

*“Generally, Social responsibility projects such as clothing, books, various tools, hospitals, nurseries, wellness training, nursing homes, first aid training, and hygiene training are carried out for families in need.” (A2).*

## Discussion and conclusion

The participants of this study consist of 15 school administrators working in primary schools affiliated with the Ministry of Education of the Turkish Republic of Northern Cyprus in the 2020–2021 academic year. In the study, the opinions of the school administrators were taken to examine their roles in school administration and improving the quality of school life through social responsibility projects. Interviews were conducted through live connections.

The study revealed that social responsibility projects have an important place in improving the quality of school life, in which the administrators have the most important tasks in this regard. They maintain the functioning of the school in good communication with students, teachers, and all staff. In addition, educational activities are made enjoyable according to the expectations and learning levels of the students and increase the need for extra-class activities. The importance of learning doing is strongly emphasized. Similarly, in research conducted by Wigmore-Álvarez and Ruiz-Lozano (2012) and Cristina et al. (2017), it was pointed out that how quality management can be a foundation for developing social responsibility. In addition, this finding of the current study is also supported by the research of Sitnikov and Bocean (2015).

For organizations to embrace ethically and socially responsible thinking, the provision needs to be “proactive,” with fundamental ethics programs taught by committed schools (Cornelius et al., 2007). Apaydın and Ercan (2010) mention on how social responsibility projects are also important for schools and in their study primary school administrators had more positive ideas about human rights, environmental problems, and business ethic than secondary school administrators did. Kelley et al. (2008) have similar findings to our study that school’s engagement in the process of practicing social responsibility and clarifying its meaning and application has made apparent the natural linkage between social responsibility and professionalism.

The participant school administrators stated that the best way to succeed in social responsibility projects is to align these activities with educational programs, carry out planned and programmed work with the teams to be created, and raise awareness by adding all stakeholders to contribute starting at an early stage. Increasing social responsibility projects in primary schools have had positive effects on the quality of school life. George et al. (2018) also indicated that to improve students’ performance, educational institutions need to build a powerful strategy to boost educational services. In addition to increasing students’ academic success, there have also been positive effects on their continuity in school.

A school whose quality of life increases always has an exemplary position (Durmaz, 2008). A planned, programmed, systematic, and coordinated order is formed in the structure and functioning of the school. A school environment that works in unity and togetherness is formed. Schools are not just a place that is separate from society and is taught in it; they are environments in which community life is experienced in a real and effective way (Dewey, 2010).

Special attention should be given to the quality of school life by administrators and teachers. It is very important to examine the quality of school life due to the relationship between both the qualifications of school life and their academic achievements. Students’ communication with their teachers and their commitment to their duties at the school are of crucial importance in school life (Yılmaz, 2005). With school quality of life, we can see students’ sense of acceptance and ownership of school, love and respect for their friends and teachers, socialization, and academic success (Erden and Erdem, 2013).

According to Argon and Demirer (2015), school administrators should determine the vision and mission of the school to increase student’s awareness of social responsibility while revealing the quality of school life; they should share the mission and vision with all stakeholders. In social responsibility projects, students and parents, as well as school administrators and teachers, should effectively maintain their contributions and support in their relations with the school environment.

To be effective, especially school administrators and teachers have important tasks. It should be noted that these planned and programmed projects motivate students against their school and lessons and lead them to success, as well as increase the quality of school life. According to Kucinska-Landwojtowicz et al. (2020), applying a process approach to the management of educational activities is possible.

## Recommendations

This study aimed to find out the system to develop the quality of social life in schools through social responsibility projects. It was found that students are excited to take part in social responsibility projects. Thus, significant improvements have been made in their academic achievement.

It can be suggested that, in a school, if there is a weakness in the quality of school life, it should be investigated considering any economic, social, or political reasons. Moreover, in-service training can be provided assuming that administrators and teachers may not be conscious enough. In the same way, conferences can be organized to raise awareness of stakeholders outside the school.

It can be further stated that, as this study was carried out before the COVID-19 pandemic, present studies will possibly be more effective to see what activities were carried out during the pandemic. Furthermore, social responsibility projects should be planned and implemented in the field of education where all stakeholders can act together. Besides these, the region and environmental conditions of the school should be taken into account in determining social responsibility projects.

## Data availability statement

The raw data supporting the conclusions of this article will be made available by the authors, without undue reservation.

## Ethics statement

The studies involving human participants were reviewed and approved by Ethical Committee Board of Near East University. The patients/participants provided their written informed consent to participate in this study.

## Author contributions

Both authors listed have made a substantial, direct, and intellectual contribution to the work, and approved it for publication.
